# *In vitro* antibiofilm and intracellular activity of delafloxacin against *Staphylococcus aureus* and *Staphylococcus epidermidis* in bone and joint infections

**DOI:** 10.1128/spectrum.02461-25

**Published:** 2026-01-13

**Authors:** Angélique Sion, Marion Martin, Mélanie Bonhomme, Jérôme Josse, Florent Valour, Frédéric Laurent, Céline Dupieux

**Affiliations:** 1 Centre International de Recherche en Infectiologie (CIRI), INSERM U1111-CNRS UMR5308-Université Lyon 1-ENS Lyon 236341, Lyon, France; 2 Service de Maladies Infectieuses et Tropicales, Hospices Civils de Lyon 26900, Lyon, France; 3 Service de Bactériologie, Centre National de Référence des Staphylocoques, Institut des Agents Infectieux, Hospices Civils de Lyon 26900, Lyon, France; Hartford Hospital, Hartford, Connecticut, USA

**Keywords:** biofilm, intracellular, bone and joint infections, delafloxacin, staphylococci

## Abstract

Staphylococcal bone and joint infections (BJIs) represent significant challenges for treatment. Fluoroquinolone-based combination therapies, especially involving levofloxacin, are pivotal antimicrobials in their management. However, resistance to levofloxacin is prevalent in methicillin-resistant strains. Delafloxacin, a novel broad-spectrum fluoroquinolone, may offer a therapeutic alternative. This study aimed to investigate the *in vitro* antibiofilm and intracellular activities of delafloxacin in a bone context. The antibiofilm activity of delafloxacin was assessed using two pairs of levofloxacin-susceptible/resistant *Staphylococcus aureus* strains (6850-S/6850-R; Clin-S/Clin-R), as well as 10 clinical strains from BJI (*S. aureus*, *n* = 5; *Staphylococcus epidermidis*, *n* = 5). The 90% minimal biofilm eradication concentration (MBEC90) of delafloxacin was compared with that of rifampicin, vancomycin, and levofloxacin after a 24-h treatment of biofilm-embedded staphylococci. Additionally, the intracellular activity of delafloxacin and comparators was evaluated using the 6850-S/6850-R and Clin-S/Clin-R strains in an intraosteoblastic (MG-63) infection model. Delafloxacin showed significant activity against levofloxacin-susceptible staphylococci in planktonic (minimum inhibitory concentration [MIC]: [0.008–0.016] mg/L) and biofilm states (MBEC90: [0.008–0.064] mg/L), and inside osteoblasts (bacterial eradication at bone concentration: −75.0% for 6850-S and −34.8% for Clin-S), but a strain-dependent activity against levofloxacin-resistant staphylococci (MIC [0.032–8] mg/L), both in biofilm (MBEC90: [0.064–>4] mg/L) and in the intracellular compartment (bacterial eradication at bone concentration: −63.6% for 6850-R and null for Clin-R). This study confirms the promising potential of delafloxacin to treat staphylococcal BJIs, but further investigations, including animal models and clinical trials, are needed to better understand its efficacy, particularly its variable effect against levofloxacin-resistant strains.

**IMPORTANCE:**

Staphylococcal bone and joint infections (BJIs) represent significant challenges for treatment. Fluoroquinolones, especially levofloxacin, play a key role in their management; however, resistance to levofloxacin is common among methicillin-resistant strains. Delafloxacin, a novel broad-spectrum fluoroquinolone, may represent a therapeutic alternative, but very limited data are available regarding its clinical utility in BJIs. In this context, our study aimed to investigate the *in vitro* antibiofilm and intracellular activities of delafloxacin in a bone context, using both levofloxacin-susceptible and levofloxacin-resistant staphylococcal strains (*Staphylococcus aureus* and *Staphylococcus epidermidis*). Our results demonstrate a significant activity of delafloxacin against levofloxacin-susceptible staphylococci both in biofilm and within osteoblasts. However, its efficacy against levofloxacin-resistant strains was variable and appeared to be strain-dependent, both in biofilm and intracellular conditions. This study confirms the promising potential of delafloxacin to treat staphylococcal BJIs, but further investigations, including animal models and clinical trials, are needed to better understand its efficacy.

## INTRODUCTION

Bone and joint infections (BJIs), particularly those related to orthopedic devices, have become increasingly prevalent, leading to significant clinical and economic impact in high-income countries (1, 2). The most commonly involved bacteria are *Staphylococcus aureus* and coagulase-negative staphylococci (CoNS) (3, 4). To treat periprosthetic staphylococcal BJIs, a combination therapy is recommended, using antibiotics with good bone diffusion, such as rifampicin and levofloxacin (5–7). While rifampicin rapidly selects resistant mutants when used in monotherapy (8, 9), fluoroquinolones, such as levofloxacin, help prevent the emergence of rifampicin resistance when used in combination (10). However, the widespread use of fluoroquinolones has led to an increasing number of resistant bacterial strains worldwide, notably in methicillin-resistant *S. aureus* (MRSA) and CoNS isolated from BJIs (11, 12).

In 2017, the FDA approved delafloxacin, a novel anionic fluoroquinolone available in oral and intravenous forms (13), which exhibits excellent *in vitro* activity against staphylococci, including MRSA; delafloxacin has equal activity against DNA gyrase and topoisomerase IV, leading to very low minimum inhibitory concentrations (MICs) (14, 15). However, several studies have reported staphylococcal isolates resistant to delafloxacin, mainly associated with double mutations in *gyr*A and *par*C (in particular at positions 80 and 84 for ParC and at positions 84 and 88 for GyrA); one study also suggested that resistance may result from the combined effect of these mutations and the presence of the plasmid-borne *qac*C efflux pump gene (15–17). Delafloxacin demonstrates strong antibiofilm activity, which is enhanced in acidic environments (18, 19), and potentially effective bone diffusion, given existing data for other fluoroquinolones (20, 21), which could make it a promising option for the treatment of BJIs (22, 23). Of note, a recent study on a collection of *Staphylococcus epidermidis* strains from BJIs found that delafloxacin was active against the large majority of ofloxacin-resistant strains using the clinical breakpoint for skin and soft tissue infections (0.25 mg/L). However, when using the breakpoint for pneumonia (0.016 mg/L), as defined for *S. aureus* by the EUCAST, less than 4% of these strains were susceptible to delafloxacin (24).

To our knowledge, there is currently very little data available on the antibiofilm and intracellular activity of delafloxacin that has been reported for staphylococci in the context of BJIs. Therefore, the present study aimed to evaluate delafloxacin antibiofilm and intracellular activity against staphylococcal strains from BJIs in biofilm and internalized inside human osteoblasts MG-63; these activities were compared to those of levofloxacin using levofloxacin-susceptible and levofloxacin-resistant strains.

## MATERIALS AND METHODS

### Bacterial strains and MIC determination

The antibiofilm and intracellular activities of the antibiotics were evaluated using two levofloxacin-susceptible (S) *S. aureus* strains isolated from BJI: a reference strain originally cultured from BJI, *S. aureus* 6850 (25), and a clinical *S. aureus* isolate obtained from a patient with recurrent BJI managed at the *Hospices Civils de Lyon* (Lyon, France), referred to as Clin. From these two susceptible strains, levofloxacin-resistant (R) mutants were obtained *in vitro,* resulting in two pairs of levofloxacin-susceptible/levofloxacin-resistant isolates (details are provided below). In addition, the antibiofilm activity was evaluated using five S. *aureus* (A1 to A5) and five *S*. *epidermidis* (E1 to E5) unrelated strains isolated from French patients with BJI; in each species, three of the five strains were levofloxacin-resistant and selected with different delafloxacin MICs. The MICs of rifampicin (Sanofi, Paris, France), vancomycin (Sandoz, Basel, Switzerland), levofloxacin (Arrow, Lyon, France), and delafloxacin (Sigma-Aldrich, Saint-Quentin-Fallavier, France) were determined using the broth microdilution method, following the 2024 guidelines of EUCAST and the French Committee for Antimicrobial Susceptibility Testing (CA-SFM/EUCAST) (26).

### Induction of levofloxacin-resistant mutants

To generate levofloxacin-susceptible/levofloxacin-resistant pairs, the two levofloxacin-susceptible *S. aureus* strains (6850-S and Clin-S) were made resistant to levofloxacin. For each strain, a 0.5 McFarland bacterial suspension was prepared in 0.85% NaCl and diluted 1:100 in Mueller-Hinton broth (MH, Sigma-Aldrich). The bacterial suspensions were then exposed to increasing concentrations of levofloxacin (0.06 to 256 mg/L) in MH, at a final volume of 10 mL. After a 24-h incubation at 37°C, the MICs of the strains were determined, and the suspension exposed to the highest concentration allowing bacterial growth (0.5× MIC) was diluted 1:10 to inoculate fresh MH containing the same range of levofloxacin concentrations. This experiment was repeated daily to increase the strains’ MIC by selecting resistant mutants and continued until the MIC reached its maximum and remained stable for three consecutive passages. The obtained levofloxacin-resistant strains (6850-R and Clin-R) were then successively subcultured five times on blood agar, and their levofloxacin MICs were determined using ETEST gradient strips (bioMérieux, Marcy l’Etoile, France).

### Whole-genome sequencing

The two levofloxacin-susceptible/levofloxacin-resistant *S. aureus* pairs and the 10 isolates used in biofilm experiments were sequenced using a NextSeq 550 (Illumina, San Diego, CA, USA) with a 2 × 150 bp paired-end strategy. Raw reads were cleaned using Trimmomatic v0.39 and Cutadapt v3.4 (27), assembled with SPAdes version v3.14.1 (28), and annotated using Bakta v1.7.0 (29). The molecular mechanisms associated with fluoroquinolone resistance (target mutations and efflux pump genes) were characterized in all isolates studied (Supplemental material). Comparative analyses between the levofloxacin-susceptible and -resistant strains were performed using Mummer4 v4.0.0rc1 (30). A single-nucleotide polymorphism study was performed using Snippy v4.6.0 to identify the mutations involved in fluoroquinolone resistance.

### Doubling time of levofloxacin-susceptible/levofloxacin-resistant pairs

The 6850 and Clin *S. aureus* strains were suspended in brain heart infusion (BHI) broth and incubated overnight at 37°C with 190 rpm agitation. The next day, the optical density (OD) at 600 nm was adjusted to 0.05. Each suspension was then placed in triplicate into a 96-well plate (Greiner, Kremsmünster, Austria) and incubated in a TECAN plate reader for 20 h at 37°C. OD_600nm_ was measured every 15 min. Growth rate during the exponential phase was calculated as µ = [ln(Y₂) – ln(Y₁)]/(T₂ – T₁), and the doubling time was determined as ln(2)/µ. Y represents the optical density (OD) at 600 nm, and T represents time.

### Eradication of biofilm-embedded *S. aureus* and *S. epidermidis*

The ability of delafloxacin to eradicate staphylococci in mature biofilms was assessed by determining the minimum biofilm eradication concentration (MBEC), meaning the MBEC that eradicates 90% (MBEC90) and 99% (MBEC99) of biofilm-embedded bacteria. The comparator antibiotics included levofloxacin, rifampicin, and vancomycin. Rifampicin was selected because of its documented efficacy against staphylococcal biofilm, while vancomycin was selected for its comparatively limited antibiofilm activity (8, 31). Strains were suspended in BHI broth and incubated overnight at 37°C with 190 rpm agitation. The following day, OD_600nm_ was adjusted to 1 (±0.05) and diluted 1:100. Bacterial cultures were then added into a non-treated cell culture 96-well plate and incubated for 24 h at 37°C without agitation to allow biofilm formation. After incubation, the supernatants were removed, and the biofilms were washed using the steam-based method for 40 min (32). The biofilms were then exposed to varying antibiotic concentrations in MH, ranging from 0.25 to 128 mg/L for rifampicin, vancomycin, and levofloxacin, and from 0.002 to 4 mg/L for delafloxacin, and incubated for 24 h at 37°C. After incubation, supernatants were removed, and the biofilms were washed using the steam-based method for 40 min. Finally, bacteria were resuspended in PBS by scraping the wells using sterile pipette tips. Bacterial counts were determined by plating serial dilutions onto Columbia agar with 5% sheep blood (COS; bioMérieux), incubated for 24 h at 37°C.

### Intracellular activity and protective effect of delafloxacin in an acute model of osteoblast infection

The efficacy of delafloxacin in eradicating *S. aureus* internalized in osteoblasts was compared to that of levofloxacin, with rifampicin and vancomycin included as positive and negative controls, respectively (8, 31). The concentrations tested were selected based on the MIC of the levofloxacin-susceptible strains (6850-S and Clin-S). Each antibiotic was tested at concentrations of 0.5×, 1×, 5×, and 10× the MIC, which included the bone concentrations (Cbone) of vancomycin (4 mg/L) and levofloxacin (5 mg/L). In addition, for delafloxacin, the estimated Cbone (1.25 mg/L) was tested (33–36).

The previously described *in vitro* osteoblast infection model was used to evaluate the effects of antibiotics on cytotoxicity and bacterial load during intracellular infection (37). Human osteoblasts from the MG63 cell line were cultured in a growth medium consisting of DMEM supplemented with 10% fetal bovine serum, with or without 1% penicillin-streptomycin (PS; 10,000 units of penicillin and 10 mg of streptomycin per milliliter). For each experiment, 24-well cell culture plates (Sarstedt, Nümbrecht, Germany) were seeded with 100,000 cells per well in the growth medium containing PS. All cell culture reagents were purchased from Gibco (Paisley, UK).

For the antibiotic intracellular activity experiments against levofloxacin-susceptible/-resistant *S. aureus* pairs, the strains were first cultured in BHI broth, incubated overnight at 37°C with 190 rpm agitation. The following day, OD_600nm_ was adjusted to 0.5 for each strain (OD/CFU correlations were previously established). These suspensions were centrifuged, the supernatants removed, and the pellets resuspended in the cell culture medium without PS. Simultaneously, three wells of osteoblast cells were counted to normalize the bacterial suspensions to a multiplicity of infection (MOI) of 100 bacteria/cell. Bacterial suspensions were then added to the osteoblasts and incubated for 2 h. Subsequently, cells were washed with PBS and incubated for 1 h with 10 mg/L lysostaphin to eliminate extracellular bacteria. Infected osteoblasts were then washed with PBS and treated with varying concentrations of antibiotics in the cell culture medium supplemented with 10 mg/L lysostaphin to kill any bacteria released into the extracellular medium during the treatment period. After 24 h of antibiotic treatment, the osteoblasts were washed with PBS and lysed with sterile water for 15 min at room temperature, followed by 15 min on ice. Dilutions of these lysates were spiral-plated (Easyspiral; Interscience, St-Nom-la-Bretèche, France) on tryptic soy agar (TSA, bioMérieux) and incubated for 24 h to quantify intracellular bacteria, using an automated colony counter (Scan 1200, Interscience). Concomitantly, cells were counted using flow cytometry (Attune NxT flow cytometer; Invitrogen, Carlsbad, CA, USA) in duplicate for each condition, allowing the determination of CFU counts relative to the actual number of cells. Infected but untreated cells served as controls in all experiments.

### Statistical analysis

Experiments were performed independently three times in triplicate, except for the screening of 10 strains used in the biofilm eradication experiments, which was performed only once. Results are expressed as means and standard deviations. Data were compared between treatment groups using a Mann-Whitney U-test with Prism software (GraphPad, San Diego, CA, USA). A *P* value <0.05 was considered significant.

## RESULTS

### Genetic and phenotypic comparison of levofloxacin-susceptible/levofloxacin-resistant *S. aureus* pairs

The two levofloxacin-susceptible strains (6850-S and Clin-S) had identical MICs for rifampicin (0.016 mg/L), vancomycin (1 mg/L), levofloxacin (0.5 mg/L), and delafloxacin (0.008 mg/L), and were susceptible to these antibiotics. Regarding their levofloxacin-resistant counterparts, the MICs were increased for levofloxacin (6850-R: 16 mg/L; Clin-R: 8 mg/L) and delafloxacin (both 6850-R and Clin-R: 0.064 mg/L), with a greater increase for levofloxacin (6850-R: 32×; Clin-R: 16×) compared to delafloxacin (both strains: 8×; Table 1). Levofloxacin-resistant mutants remained susceptible to rifampicin and vancomycin.

**TABLE 1:** Rifampicin, vancomycin, levofloxacin, and delafloxacin MICs and MBEC90/99 for the two levofloxacin-susceptible/-resistant *S. aureus* pairs (6850 and Clin) and the 10 screening BJI strains tested (*S*. *aureus, n* = 5; *S*. *epidermidis, n* = 5)^*a*^

	Rifampicin			Vancomycin			Levofloxacin			Delafloxacin		
	MIC (mg/L)	MBEC (mg/L)		MIC (mg/L)	MBEC (mg/L)		MIC (mg/L)	MBEC (mg/L)		MIC (mg/L)	MBEC (mg/L)	
Strain		90	99		90	99		90	99		90	99
6850-S (MSSA)	0.016	<0.25	64	1	8	>128	0.5	0.5	1	0.008	0.016	0.25
6850-R (MSSA)	0.032	<0.25	>128	1	4	>128	16	32	>128	0.064	0.064	1
Clin-S (MSSA)	0.016	<0.25	>128	1	>128	>128	0.5	2	>128	0.008	0.064	2
Clin-R (MSSA)	0.032	<0.25	>128	1	64	>128	8	32	>128	0.064	0.5	2
A1-S (MSSA)	0.016	<0.25	>128	1	16	>128	0.5	1	32	0.008	0.032	0.064
A2-S (MRSA)	0.016	<0.25	64	1	8	>128	0.5	0.5	64	0.008	0.032	0.25
A3-R (MRSA)	0.016	<0.25	64	1	>128	>128	8	>128	>128	0.032	4	>4
A4-R (MSSA)	0.016	32	128	1	>128	>128	128	>128	>128	4	>4	>4
A5-R (MRSA)	0.016	<0.25	8	1	8	>128	32	128	>128	0.5	2	>4
E1-S (MSSE)	0.032	<0.25	<0.25	2	16	16	0.5	<0.25	0.5	0.016	0.008	0.032
E2-S (MRSE)	0.016	<0.25	32	2	8	>128	1	2	4	0.016	0.064	0.125
E3-R (MRSE)	0.016	<0.25	<0.25	2	16	16	32	64	64	0.5	0.5	2
E4-R (MRSE)	0.008	<0.25	64	2	16	>128	8	>128	>128	0.25	>4	>4
E5-R (MRSE)	>4	>128	>128	2	16	>128	>128	>128	>128	8	>4	>4

^*a*^ MIC, minimal inhibitory concentration; MBEC90/99, minimum antibiotic concentration that resulted in a ≥90%/≥99% reduction in viable biofilm-embedded bacteria compared to the untreated control group after 24 h of treatment. MSSA, methicillin-susceptible *S. aureus*; MRSA, methicillin-resistant *S. aureus*; MSSE, methicillin-susceptible *S. epidermidis*; MRSE, methicillin-resistant *S. epidermidis*. Strain names end in -S or -R, depending on their susceptibility/resistance to levofloxacin. EUCAST 2024 breakpoints are 1 mg/L for levofloxacin, and 0.016 and 0.25 mg/L for delafloxacin, for community-acquired pneumonia and skin and skin structure infections, respectively.

The effect of the acquisition of fluoroquinolone resistance on the fitness of levofloxacin-resistant strains was then investigated. No significant difference in doubling time was observed for the 6850-S/6850-R pair, as opposed to the Clin-S/Clin-R pair (44.1 vs 50.8 min; *P* = 0.0014; Table 2).

**TABLE 2:** Fitness and genomic comparison of the two levofloxacin-susceptible/-resistant *S. aureus* pairs used in the study^*a*^^,*b*^

*S. aureus* strain	Doubling time (min; mean ± standard deviation)	Mutations conferring resistance to fluoroquinolones		
		Gene	Product	Effect
6850-S	50.6 ± 4.8	NA	NA^*c*^	NA
6850-R	49.4 ± 3.1 (N.S vs 6850-S)	*par*C	DNA topoisomerase IV subunit A	c.239C>T (S80F) and c.251A>G (E84G)
		*gyr*B	DNA gyrase subunit B	c.1309G>A (D437N)
Clin-S	44.1 ± 5.6	NA	NA	NA
Clin-R	50.8 ± 6.5 (*P* = 0.0014 vs Clin-S)	*par*C	DNA topoisomerase IV subunit A	c.250G>A (E84G)
		*gyr*A	DNA gyrase subunit A	c.900_905delTGGTGT (G301_V302del)
		*gyr*B	DNA gyrase subunit B	c.1366C>T (P456S)

^*a*^ The levofloxacin-susceptible strains were used as a reference for comparison with the resistant strains.

^*b*^ *parC*, DNA topoisomerase IV subunit A; *gyrA*, DNA gyrase subunit A; *gyrB*, DNA gyrase subunit B.

^*c*^ NA, not applicable.

Finally, mutations in levofloxacin-resistant strains were characterized using whole-genome sequencing (WGS). Strain 6850-R harbored three mutations directly involved in fluoroquinolone resistance: two substitutions in the DNA topoisomerase IV subunit A (Ser-80→Phe; Glu-84→Gly) and one substitution in the DNA gyrase subunit B (Asp-437→Asn). Strain Clin-R also had three mutations conferring fluoroquinolone resistance: a substitution in the DNA topoisomerase IV subunit A (Glu-84→Lys), a disruption in the DNA gyrase subunit A (Gly-301_Val-302del), and a substitution in the DNA gyrase subunit B (Pro-456→Ser; Table 2). In both levofloxacin-resistant strains, other mutations not involved in fluoroquinolone resistance were observed, including insertions in FdhF and SarS proteins in Clin-R (Tables S1 and S2).

### Antibiofilm activity of delafloxacin and comparators

The majority of strains (12/14) exhibited low MBEC90 values for rifampicin (<0.25 mg/L; Table 1), except the rifampicin-susceptible *S. aureus* strain A4 (32 mg/L) and the rifampicin-resistant *S. epidermidis* strain E5 (>128 mg/L). However, 12/14 strains presented high MBEC99 values for this antibiotic (≥64 mg/L). For vancomycin, all strains had high MBEC90 ([4;>128] mg/L) and MBEC99 values ([16;>128] mg/L) despite being susceptible to vancomycin according to the MIC.

Regarding fluoroquinolones, the increase in MBEC90 in 6850-R and Clin-R, as compared to their levofloxacin-susceptible counterparts, was lower for delafloxacin (6850-R: 4×; Clin-R: 8×) than for levofloxacin (6850-R: 64×; Clin-R: 16×; Table 1). A similar pattern was observed for MBEC99 in the 6850-S/6850-R pair, but not in the Clin-S/Clin-R pair, which did not exhibit an increase in MBEC99 for both antibiotics. For the 10 clinical strains isolated from BJIs, delafloxacin had good antibiofilm activity against levofloxacin-susceptible strains (MBEC90 [0.008;0.064] mg/L; MBEC99: [0.032;0.25] mg/L), but the MBEC90 and MBEC99 were higher for levofloxacin-resistant strains (MBEC90: [0.5;>4] mg/L; MBEC99: [2;>4] mg/L). A similar pattern was observed among *S. aureus* and *S. epidermidis* strains. However, delafloxacin antibiofilm activity was found to be relatively strain-dependent; for *S. epidermidis* strain E3 (delafloxacin MIC: 0.5 mg/L) the MBEC90 was 0.5 mg/L (1× MIC) and the MBEC99 was 2 mg/L (4× MIC), whereas for strain E4 (delafloxacin MIC: 0.25 mg/L) the MBEC90 was >4 mg/L (>16× MIC) and the MBEC99 was >4 mg/L (>16× MIC). Similar results were observed in *S. aureus* strains for A3 (delafloxacin MIC: 0.032 mg/L; MBEC90: 4 mg/L, i.e., 125× MIC) and A5 (delafloxacin MIC: 1 mg/L; MBEC90: 2 mg/L, i.e., 2× MIC). WGS characterization of the molecular mechanisms associated with fluoroquinolone resistance in all isolates revealed the presence of various combinations of target mutations and very few plasmidic efflux pumps (except *qac*C in A5, and *qac*A in E4 and E5) (Table S3).

The efficacy of the four antibiotics at or near theoretical Cbone was also determined for all strains (Table S4). As expected, rifampicin was effective against all strains, except strain E5 (rifampicin-resistant). Conversely, vancomycin had poor activity against most strains. Both fluoroquinolones, levofloxacin and delafloxacin, demonstrated good efficacy against levofloxacin-susceptible strains at Cbone. However, against levofloxacin-resistant strains, delafloxacin demonstrated greater efficacy than levofloxacin, except for strain A4, for which the two antibiotics had similar activity.

### Intraosteoblastic activity and protective effect of levofloxacin and delafloxacin against *S. aureus*

**Fig 1:**
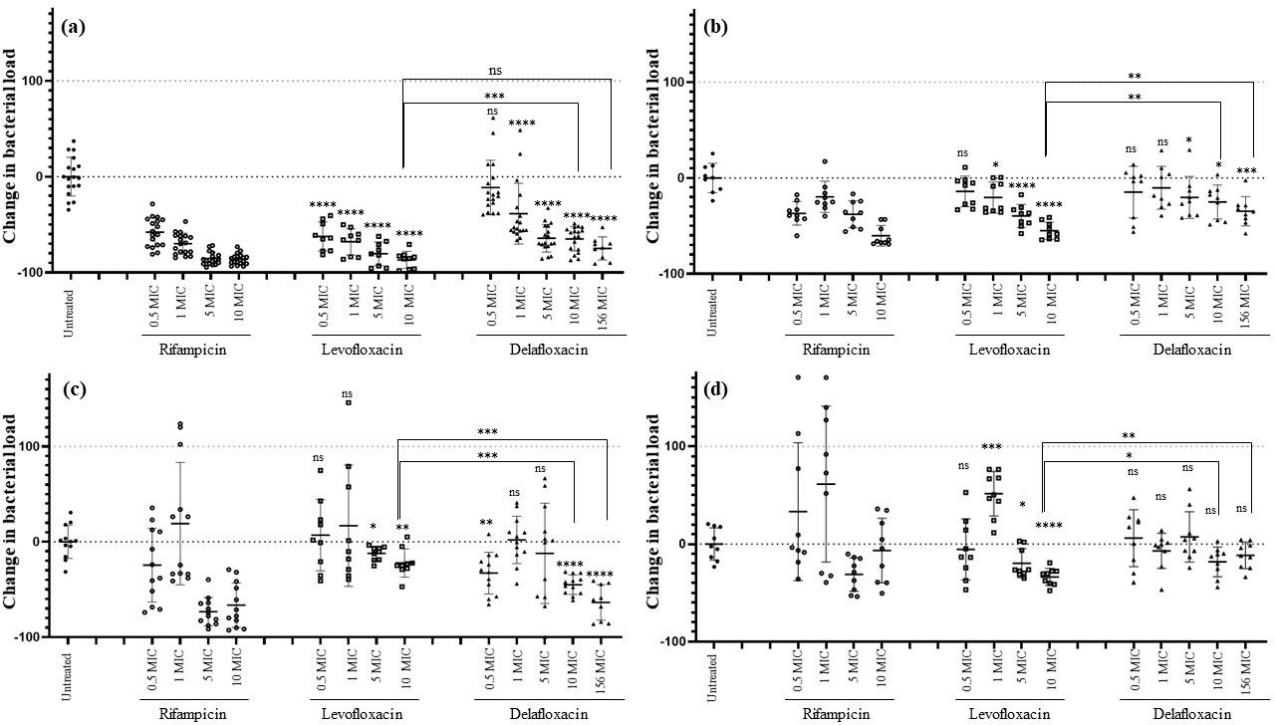
Intracellular activity of rifampicin, levofloxacin, and delafloxacin during a 24 h treatment on MG-63 cells infected with levofloxacin-susceptible *S. aureus* strains and their levofloxacin-resistant counterparts. Osteoblasts were infected at MOI 100 with the two levofloxacin-susceptible/-resistant *S. aureus* pairs (**a**: 6850-S; **b**: Clin-S; **c**: 6850-R; **d**: Clin-R) and treated for 24 h at 0.5×, 1×, 5×, and 10× the MIC for each antibiotic and 156× the MIC for delafloxacin (bone concentrations of levofloxacin and delafloxacin corresponded to 10× and 156× the MIC, respectively). Results are presented as mean and standard deviation (each condition was tested in triplicate in three independent experiments). The change in bacterial load for each condition was normalized to the untreated condition (equal to zero reduction percentage). Mann-Whitney U-test (*P < 0.05; **P < 0.01; ***P < 0.001, ****P < 0.0001) was used to determine the difference between treated and untreated cells for levofloxacin and delafloxacin, and the difference between cells treated with levofloxacin or delafloxacin (it should be noted that not all statistics are shown for clarity).

Rifampicin was highly effective in reducing the intracellular inoculum after 24 h of treatment for both levofloxacin-susceptible strains (6850-S: −86.1% ± 5.8% and Clin-S: −60.1% ± 10.8% at 10× MIC, *P* vs untreated: <0.0001 for both). However, its activity was lower against both levofloxacin-resistant strains (6850-R: −66.6% ± 23.4% at 10× MIC, *P* vs untreated: <0.0001; Clin-R: −6.7% ± 33.1% at 10× MIC, *P* vs untreated = 0.71; Fig. 1). Regarding vancomycin, it was not effective on either levofloxacin-susceptible or levofloxacin-resistant strains (data not shown).

Regarding fluoroquinolones, for the 6850-S/6850-R pair, levofloxacin (at Cbone/10× MIC: −86.9% ± 8.8%) was more effective than delafloxacin (at 10× MIC: −64.9% ± 12.8%, *P* = 0.0003; at Cbone/156× MIC: −75.0% ± 12.1%, *P* = 0.0503) on the levofloxacin-susceptible strain. Conversely, in the 6850-R strain, levofloxacin (at Cbone/10× MIC: −22.1% ± 14.8%) was less effective compared to delafloxacin (at 10× MIC: −45.1% ± 9.9%, *P* = 0.0003; at Cbone/156× MIC: −63.6% ± 18.7%, *P* = 0.0003). For the Clin-S/Clin-R pair, levofloxacin (Cbone/10× MIC: −55.0% ± 9.1%) was more effective than delafloxacin (at 10× MIC: −25.2% ± 17.7%, *P* = 0.0019; at Cbone/156× MIC: −34.8% ± 15.2%, *P* = 0.0019) on the levofloxacin-susceptible strain. However, for the levofloxacin-resistant strain, levofloxacin (at Cbone/10× MIC: −33.7% ± 8.8%) remained effective, while delafloxacin had reduced activity (at 10× MIC: −18.2% ± 15.2%, *P* = 0.0315; at Cbone/156× MIC: −11.7% ± 13.6%, *P* = 0.0019; Fig. 1).

The two pairs tested were cytotoxic to MG-63 osteoblasts (uninfected cells vs untreated infected cells: *P* < 0.005 for all). The cytotoxicity of 6850-S was significantly greater than that of Clin-S (*P* = 0.0001). However, for both pairs, the antibiotic concentrations tested did not significantly protect MG-63 cells from bacterial cytotoxicity, although certain concentrations had high bacterial eradication (Fig. S1).

## DISCUSSION

The present study reported that delafloxacin was effective against levofloxacin-susceptible *S. aureus* and *S. epidermidis* in biofilm and exhibited a strain-dependent activity against levofloxacin-resistant strains in biofilm. In addition, the intracellular activity of delafloxacin against *S. aureus* was also strain-dependent, even on levofloxacin-susceptible strains.

The antibiofilm activity of levofloxacin and delafloxacin was compared using two pairs of levofloxacin-susceptible/-resistant *S. aureus* strains (a reference strain and a clinical strain), and 10 clinical strains (*S. aureus* and *S. epidermidis*), all isolated from BJIs. Delafloxacin led to a lower increase in MBEC90 compared to levofloxacin in levofloxacin-susceptible/levofloxacin-resistant pairs, which is likely due to delafloxacin’s unique chemical properties. Unlike previous fluoroquinolones, which are zwitterions, delafloxacin presents an anionic form at neutral pH and a neutral form at acidic pH, improving lipid membrane diffusion, especially in acidic environments, such as some biofilms (21, 38). Of note, a previous study demonstrated delafloxacin’s greater bactericidal activity and higher accumulation in bacteria in acidic conditions (18, 19). Our results report a similar antibiofilm activity of delafloxacin in *S. aureus* and *S. epidermidis* isolates. Its activity against levofloxacin-resistant strains was strain-dependent, as already described in delafloxacin and various antibiotics, for both reference and clinical strains (20, 38). For example, Bauer et al*.* noted that at equivalent concentrations, delafloxacin had greater antibiofilm activity against MRSA than MSSA (20). Differences in delafloxacin efficacy on biofilm could also be attributed to variations in biofilm composition or to the amount of biofilm formed; a study found that delafloxacin penetration decreased when polysaccharide content increased in the biofilm matrix (38).

The activity of levofloxacin and delafloxacin was assessed on levofloxacin-susceptible/-resistant *S. aureus* pairs internalized in human osteoblasts (MG63 lineage) using a reference and a clinical strain. Notably, all tested antibiotics were less effective against the levofloxacin-susceptible clinical strain (Clin-S) than against the reference strain (6850), despite similar MICs. This reduced efficacy could be attributed to the isolation of the Clin-S strain from a patient with recurrent BJI, suggesting an adaptive or evolutionary trade-off that may confer increased antibiotic tolerance and altered intracellular behavior, particularly regarding its subcellular localization dynamics and the pH environment in which it resides (39). Our study also showed that levofloxacin was more effective than delafloxacin against levofloxacin-susceptible strains. This may reflect differences in bone cell penetration and intracellular accumulation in our model. Notably, to our knowledge, no data are currently available on the bone penetration of delafloxacin in humans. Furthermore, this discrepancy may be related to the subcellular localization of bacteria within osteoblasts, as delafloxacin appears less active when bacteria reside in the cytosol rather than in the lysosome, which has an acidic pH. While delafloxacin showed good intracellular activity against the levofloxacin-susceptible strains in both pairs, this activity was strain-dependent on levofloxacin-resistant mutants. Delafloxacin was effective against 6850-R at 10× MIC and Cbone, unlike levofloxacin, but was less effective against Clin-R, in which levofloxacin remained active at 10× MIC. A study by Lemaire et al*.* previously demonstrated greater intracellular activity of delafloxacin compared to moxifloxacin against *S. aureus* ATCC25923 in THP-1 macrophages, but did not include clinical strains (19). The strain-dependent activity may be explained by genetic differences: 6850-R has common mutations in ParC and GyrB, whereas Clin-R has a common mutation in ParC but also a GyrA disruption, as well as a mutation in GyrB, which are less frequently reported (40). Unlike levofloxacin, which primarily targets topoisomerase IV, delafloxacin targets both topoisomerase IV and DNA gyrase. Therefore, mutations in these two targets can reduce delafloxacin activity, as observed for the Clin-R strain. Additionally, Clin-R exhibited a significant fitness cost, illustrated by a slower growth compared to Clin-S, which may contribute to increased antibiotic tolerance. Previous studies have shown that the development of fluoroquinolone resistance can compromise the viability of bacterial strains, including staphylococci (41, 42). This slower growth, associated with fluoroquinolone resistance mutations, was not observed in the 6850 pair.

The clinical strain Clin-S/Clin-R included herein is interesting since it allowed us to show that delafloxacin can present with a good antibiofilm activity and a reduced intracellular efficacy against the same strain. Of note, the good antibiofilm activity of delafloxacin in Clin-R was not found in most of the other included levofloxacin-resistant strains. It is possible that the mutations in the *fdh*F (formate dehydrogenase) and *sar*S (virulence factor repressor) genes may affect biofilm formation and composition in this strain. Studies have demonstrated that the *fdh*F gene is overexpressed in *S. aureus* biofilms (43), while mutations in the *sar*A gene, a homolog of *sar*S, have been associated with reduced biofilm formation (44, 45). If these mutations affect Clin-R biofilm, it could explain why delafloxacin remains effective against the biofilm despite the mutations in the *par*C and *gyr*A genes.

Finally, the present study demonstrated promising potential regarding the use of delafloxacin in staphylococcal BJIs, including some levofloxacin-resistant strains. However, several limitations need to be addressed. Delafloxacin bone concentration was estimated by assuming that delafloxacin has the same bone/plasma ratio as found in other fluoroquinolones, since it has never been directly measured in animals or humans. Therefore, pharmacokinetic studies are needed to confirm delafloxacin concentration in bone tissue. Further investigation is warranted using levofloxacin-susceptible/-resistant strain pairs isolated from patients with BJI, as the current findings are based on *in vitro*-derived pairs. Regarding delafloxacin antibiofilm activity, its evaluation on mature biofilms formed on prosthetic materials will be necessary, as biofilm adhesion and composition are significantly influenced by the material used (46, 47). To assess intracellular activity, this study used an osteoblast cancer line; however, using primary cells from patients would be beneficial. In contrast, investigating intracellular activity in *S. epidermidis* levofloxacin-susceptible/levofloxacin-resistant pairs may have limited relevance, as this species demonstrates minimal internalization in osteoblasts (48).

In conclusion, this study demonstrates the good antibiofilm and intracellular activity of delafloxacin against levofloxacin-susceptible strains, but strain-dependent activity against levofloxacin-resistant strains, which remains to be elucidated. To support the potential use of delafloxacin to treat staphylococcal BJIs, further *in vivo* studies, including animal models and clinical trials, are essential, especially for levofloxacin-resistant strains.

## Data Availability

All relevant materials and data supporting the findings of this study are contained within the paper. More detailed data are available from the corresponding author upon reasonable request. Sequencing reads from the 14 staphylococcal isolates included in the present study have been deposited in the European Nucleotide Archive under the accession number PRJEB98851.

## SUPPLEMENTAL MATERIAL

Supplemental material spectrum.02461-25-s0001.docx Supplemental materials and methods, Tables S1 to S4, and Figure S1.

ASM does not own the copyrights to Supplemental Material that may be linked to, or accessed through, an article. The authors have granted ASM a non-exclusive, world-wide license to publish the Supplemental Material files. Please contact the corresponding author directly for reuse.
