# Supplementary material for: In vitro antibiofilm and intracellular activity of delafloxacin against Staphylococcus aureus and Staphylococcus epidermidis in bone and joint infections
Source: Microbiol Spectr. 2026 Jan 13;14(2):e02461-25. doi: 10.1128/spectrum.02461-25 (PMC12889017; doi:10.1128/spectrum.02461-25)
Supplement: Supplemental material — s and methods, Tables S1 to S4, and Figure S1. [file spectrum.02461-25-s0001.docx]

**Supplementary data**

***In vitro* antibiofilm and intracellular activity of delafloxacin against *Staphylococcus aureus* and *Staphylococcus* *epidermidis* in bone and joint infections**

Angélique SION^1^, Marion MARTIN^1^, Mélanie BONHOMME^1^, Jérôme JOSSE^1^, Florent VALOUR^1,2^, Frédéric LAURENT^1,3^, Céline DUPIEUX^1,3^

^1^ Centre International de Recherche en Infectiologie (CIRI), INSERM U1111-CNRS UMR5308-Université Lyon 1-ENS Lyon, F-69007 Lyon, France

^2^ Hospices Civils de Lyon, Service de Maladies Infectieuses et Tropicales, F-69000 Lyon, France

^3^ Hospices Civils de Lyon, Service de Bactériologie, Centre National de Référence des Staphylocoques, Institut des Agents Infectieux, F-69000 Lyon, France

**Supplementary materials and methods (WGS analysis)**

All strains (*Staphylococcus aureus*, *n* = 9; *Staphylococcus epidermidis*, *n* = 5) were sequenced using the NextSeq 550 platform (Illumina, San Diego, CA, USA) with a 2 × 150 bp paired-end strategy. Raw reads were quality-filtered using Trimmomatic v0.39 and Cutadapt v3.4, assembled with SPAdes v3.14.1, and annotated using Bakta v1.7.0.

For the 6850-S/R and Clin-S/R strain pairs, comparative genomic analyses between levofloxacin-susceptible and -resistant isolates were performed using MUMmer4 v4.0.0rc1. Single nucleotide polymorphism (SNPs) identification was carried out with Snippy v4.6.0 to detect mutations associated with fluoroquinolone resistance.

For the 10 clinical strains used in biofilm experiments, the reference genome *S. aureus* ASM1342v1 (NCBI) was used for comparison with the *S. aureus* isolates A1–A5, while *S. epidermidis* ATCC12228 served as the reference for *S. epidermidis* isolates E1–E5. Protein sequences of each gene were retrieved from both reference and sample genomes and aligned using MAFFT (web tool) for SNP analysis.

The presence or absence of efflux pump genes was evaluated by comparing all strains with efflux pump protein sequences obtained from the UniProt database, except for LmrS and MdeA, whose sequences were retrieved from CARD. Protein sequence similarity searches were performed using BLASTp, with gene presence defined as ≥90% coverage and ≥85% identity.

**Supplementary table 1.** WGS comparison of the levofloxacin-susceptible/levofloxacin-resistant 6850-S/6850-R *S. aureus* pair.

| 6850-R contig | Pos | Type | Ref | Alt | Ftype | Effect | Gene | Product |
| --- | --- | --- | --- | --- | --- | --- | --- | --- |
| Contig 2 | 270647 | SNP | T | A |  |  |  |  |
| Contig 3 | 30762 | SNP | C | T | CDS | missense variant c.1309G>A  Asp-437→Asn | *gyrB* | DNA topoisomerase (ATP-hydrolyzing) subunit B |
| Contig 4 | 18217 | del | AT | A | CDS | frameshift variant c.225delT  Tyr-75fs | *lysR* | DNA-binding transcriptional regulator, LysR family |
| Contig 4 | 129385 | SNP | N | T | gap |  |  |  |
| Contig 4 | 182818 | SNP | C | T | CDS | missense variant c.1394C>T  Pro-465→Leu | *pTR2* | Dipeptide/tripeptide permease |
| Contig 5 | 51841 | complex | CGAC | TGAT | CDS | synonymous variant c.2151_2154delCGACinsTGAT p.719 | *sdrC* | MSCRAMM family adhesin SdrC |
| Contig 5 | 51850 | SNP | T | C | CDS | synonymous variant c.2160T>C  Asp-720→Asp | s*drC* | MSCRAMM family adhesin SdrC |
| Contig 6 | 52103 | SNP | A | G |  |  |  |  |
| Contig 6 | 59527 | SNP | C | T | CDS | missense variant c.239C>T  Ser-80→Phe | *parC* | DNA topoisomerase IV subunit A |
| Contig 6 | 59539 | SNP | A | G | CDS | missense variant c.251A>G  Glu-84→Gly | *parC* | DNA topoisomerase IV subunit A |

The levofloxacin-susceptible strain was used as a reference for comparison with the resistant strain. Pos: position; Ref: reference; Alt: alteration; SNP: single nucleotide polymorphisms; del: deletion; CDS: coding DNA sequence.

**Supplementary table 2.** WGS comparison of the levofloxacin-susceptible/levofloxacin-resistant Clin-S/Clin-R *S. aureus* pair.

| Clin-R contig | Pos | Type | Ref | Alt | Ftype | Effect | Gene | Product |
| --- | --- | --- | --- | --- | --- | --- | --- | --- |
| Contig 1 | 19174 | dup | T | TCC | CDS | frameshift variant c.113_114dupGG Thr-39fs | *moaE* | Molybdopterin synthase catalytic subunit |
| Contig 1 | 53900 | dup | A | ACG | CDS | frameshift variant c.1823_1824dupCG  Tyr-609fs | *fdhF* | Formate dehydrogenase subunit alpha |
| Contig 1 | 132081 | SNP | N | C | gap |  |  |  |
| Contig 2 | 7856 | SNP | T | A | CDS | synonymous variant  c.300A>T  Thr-100→Thr | *fur* | Ferric uptake regulation protein |
| Contig 3 | 219269 | SNP | C | T | CDS | missense variant c.250G>A  Glu-84→Lys | *parC* | DNA topoisomerase IV subunit A |
| Contig 4 | 209224 | SNP | C | T | CDS | missense variant c.1366C>T  Pro-456→Ser | *gyrB* | DNA topoisomerase (ATP-hydrolyzing) subunit B |
| Contig 4 | 210728 | del | CTGGTGT | C | CDS | disruptive inframe del c.900_905delTGGTGT  Gly-301_Val-302del | *gyrA* | DNA gyrase subunit A |
| Contig 8 | 54781 | dup | T | TG | CDS | frameshift variant c.389dupC  Asp-132fs | *sarS* | HTH-type transcriptional regulator SarS |
| Contig 11 | 34553 | complex | C | CGCCGGCAAGGT | ncRNA | inter region  n.34553_34554  insGCCGGCAAGGT | *S35* | *Staphylococcus* sRNA 35 (srn_0335) |
| Contig 22 | 1879 | ins | T | TG |  |  |  |  |
| Contig 30 | 23483 | ins | A | ACG |  |  |  |  |

The levofloxacin-susceptible strain was used as a reference for comparison with the resistant strain. Pos: position; Ref: reference; Alt: alteration; dup: duplication; SNP: single nucleotide polymorphisms; del: deletion CDS: coding DNA sequence; ins: insertion.

**Supplementary table 3.** Characterisation of the molecular mechanisms associated with fluoroquinolone resistance (target mutations and efflux pump genes) in the isolates studied using WGS analysis.

| **Strain** | **Efflux pumps** | | | | | | | | | | | | | **Mutations associated with fluoroquinolone resistance** | | | |
| --- | --- | --- | --- | --- | --- | --- | --- | --- | --- | --- | --- | --- | --- | --- | --- | --- | --- |
|  | LmrS | MepA | QacA | QacB | QacC | QacG | QacJ | MdeA | NorA | NorB | NorC | SdrM | SepA | ParC | ParE | GyrA | GyrB |
| 6850-S (MSSA) | x | x |  |  |  |  |  | x | x |  | x | x | x |  |  |  |  |
| 6850-R (MSSA) | x | x |  |  |  |  |  | x | x |  | x | x | x | S80F  E84G |  |  | D437N |
| Clin-S (MSSA) | x | x |  |  |  |  |  | x | x | x | x | x | x |  |  |  |  |
| Clin-R (MSSA) | x | x |  |  |  |  |  | x | x | x | x | x | x | E84G |  | G301_V302del | P456S |
| A1-S (MSSA) | x | x |  |  |  |  |  | x | x | x | x | x | x | V590I  V656I | I72T  E422D  E596D | E815D | E182D |
| A2-S (MRSA) | x | x |  |  |  |  |  | x | x | x | x | x | x | Y410F | N139S | A457T | Q66K |
| A3-R (MRSA) | x | x |  |  |  |  |  | x | x | x | x | x | x | S80F  Y410F | N139S | E88K  A457T | Q66K |
| A4-R (MSSA) | x | x |  |  |  |  |  | x | x | x | x | x | x | S80F  E84K  Y410F | N139S | S84L  A457T | Q66K |
| A5-R (MRSA) | x | x |  |  | x |  |  | x | x | x | x | x | x | S80F  E84K  Y410F | N139S | S84L  A457T | Q66K |
| E1-S (MSSE) |  |  |  |  |  |  |  |  |  |  |  |  |  | K272R | A87V |  |  |
| E2-S (MRSE) |  |  |  |  |  |  |  |  |  |  |  |  |  | K272R | D264Y  R302- |  |  |
| E3-R (MRSE) |  |  |  |  |  |  |  |  |  |  |  |  |  | S80F  D84Y  K272R |  | S84Y |  |
| E4-R (MRSE) |  |  | x |  |  |  |  |  |  |  |  |  |  | S80Y  K272R |  | S84F |  |
| E5-R (MRSE) |  |  | x |  |  |  |  |  |  |  |  |  |  | S80F  D84Y  K272R |  | S84Y  S85P |  |

The levofloxacin-susceptible strain was used as a reference for comparison with the resistant strain for the levofloxacin-susceptible/levofloxacin-resistant pairs. For the 10 clinical strains used in biofilm experiments, the reference genome of *S. aureus* ASM1342v1, available on NCBI, was employed for comparison with the *S. aureus* strains (n=5; A1 to A5), and the genome of *S. epidermidis* ATCC12228 was used for comparison with the *S. epidermidis* strains (n=5; E1 to E2). With regard to the presence/absence of efflux pumps, a comparison was made between all strains and protein sequences obtained from the UniProt database, with the exception of the protein sequences of LmrS and MdeA, which were obtained from CARD.

**Supplementary table 4.** *In vitro* antibiofilm activity of rifampicin, vancomycin, levofloxacin, and delafloxacin, expressed as percentage of bacterial reduction after a 24h-treatment at or near bone concentration (Cbone), against 14 staphylococcal strains.

|  | Bacterial reduction in biofilm (%)^a^ | | | |
| --- | --- | --- | --- | --- |
|  | Rifampicin | Vancomycin | Levofloxacin | Delafloxacin |
|  | Cbone  (6 mg/L) | Cbone  (4 mg/L) | Cbone  (5 mg/L) | Cbone  (1.25 mg/L) |
| Tested concentrations  close to Cbone | 4-8 mg/L | 4 mg/L | 4-8 mg/L | 1-2 mg/L |
| 6850-S (MSSA) | 96.5%-98.8% | 74.0% | 99.2%-99.7% | 99.9%-100.0% |
| 6850-R (MSSA) | 96.6%-96.3% | 97.0% | 51.8%-63.4% | 99.6%-99.2% |
| Clin-S (MSSA) | 93.9%-93.3% | 56.9% | 99.3%-97.4% | 98.1%-99.4% |
| Clin-R (MSSA) | 95.6%-93.2% | 67.3% | 22.3%-46.0% | 96.6%-97.3% |
| A1-S (MSSA) | 95.7%-91.4% | 18.8% | 96.6%-99.6% | 99.9%-100.0% |
| A2-S (MRSA) | 97.7%-97.3% | 55.3% | 97.5%-99.0% | 99.6%-99.8% |
| A3-R (MRSA) | 98.6%-96.4% | 40.0% | 0%-68.0% | 82.4%-84.9% |
| A4-R (MSSA) | 85.4%-94.5% | 54.0% | 22.0%-28.0% | 20.0%-31.0% |
| A5-R (MRSA) | 97.5%-99.8% | 76.6% | 53.7%-57.2% | 83.1%-95.5% |
| E1-S (MSSE) | 99.9%-99.5% | 0% | 99.1%-99.6% | 99.9%-99.9% |
| E2-S (MRSE) | 99.9%-99.8% | 42.2% | 99.9%-100.0% | 100.0%-100.0% |
| E3-R (MRSE) | 99.6%-99.9% | 0% | 5.6%-10.3% | 97.5%-99.9% |
| E4-R (MRSE) | 95.4%-99.4% | 43.2% | 59.5%-73.7% | 81.0%-98.0% |
| E5-R (MRSE) | 57.2%-15.1% | 4.1% | 0%-0% | 0%-54.2% |

^a^ reduction in viable biofilm-embedded bacteria compared to the untreated control group after 24 h of treatment.

MSSA: methicillin-susceptible *S. aureus*; MRSA: methicillin-resistant *S. aureus*; MSSE: methicillin-susceptible *S. epidermidis*; MRSE: methicillin-resistant *S. epidermidis*. Strain names end in -S or -R depending on their susceptibility/resistance to levofloxacin.

**Supplementary figure 1.** Evaluation of the protective effect of rifampicin, levofloxacin, and delafloxacin during a 24h-treatment on MG-63 cells infected with levofloxacin-susceptible *S. aureus* strains and their levofloxacin-resistant counterparts.


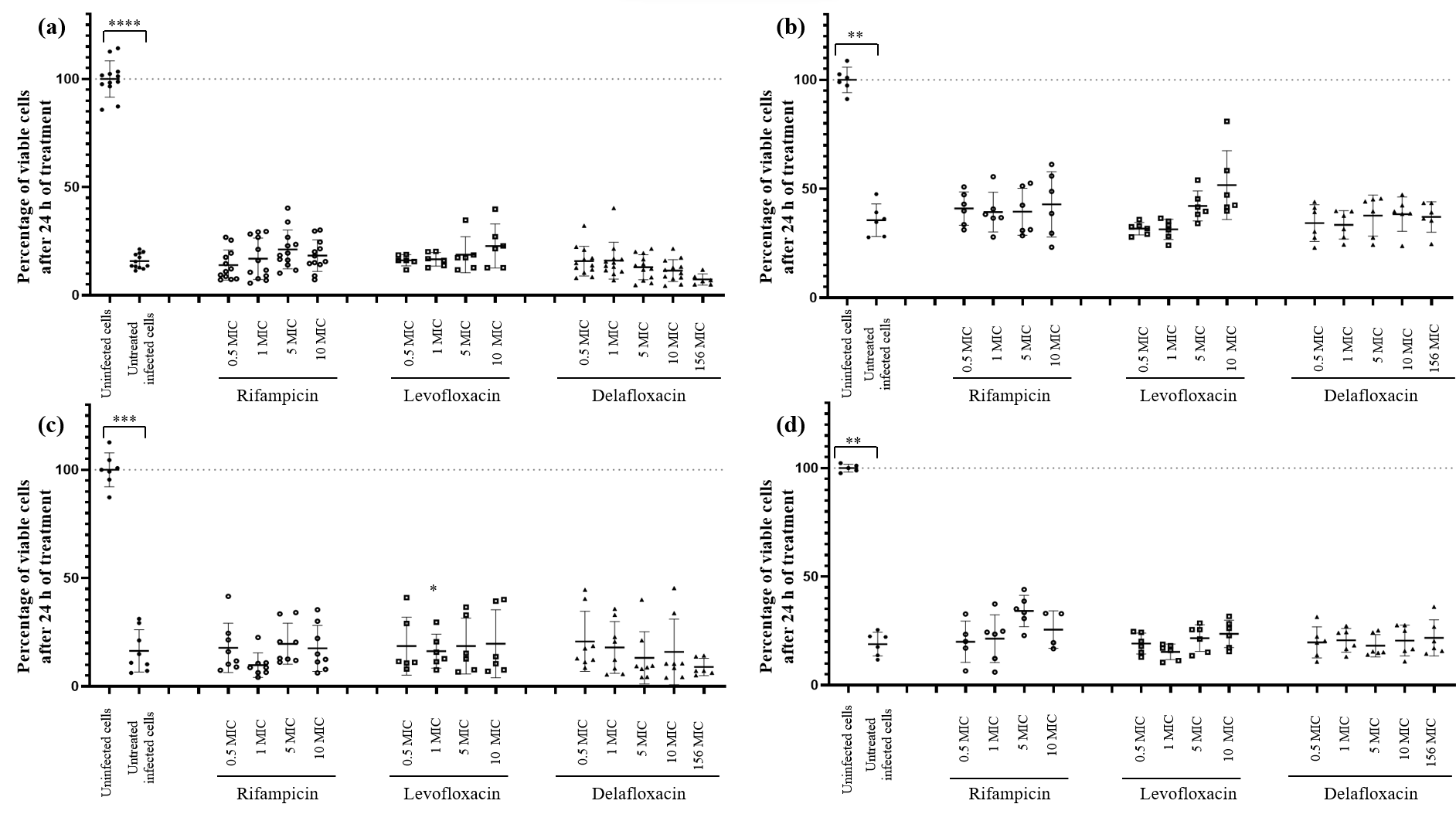


Osteoblasts were infected at MOI 100 with the two levofloxacin-susceptible/-resistant *S. aureus* pairs (a: 6850-S; b: Clin-S; c: 6850-R; d: Clin-R) and treated for 24 h at 0.5x, 1x, 5x and 10x the MIC for each antibiotic and 156x the MIC for delafloxacin. Results are presented as mean and standard deviation (each condition was tested in triplicate in three independent experiments). The percentage of viable cells for each condition was normalised to the untreated condition. Mann-Whitney U-test (*p < 0.05; **p < 0.01; ***p < 0.001, **** p < 0.0001) was used to determine the difference between uninfected and infected untreated cells and the difference between infected untreated cells and infected cells treated with rifampicin, levofloxacin, or delafloxacin (only statistically significant differences are represented on the graphs).
